# Utility of Field Tests for Predicting Cardiorespiratory Fitness and Prescribing Exercise Intensity in Cardiac Rehabilitation Programs: A Randomized Crossover Trial

**DOI:** 10.3390/jcdd13030114

**Published:** 2026-03-03

**Authors:** Blake E. G. Collins, Brett A. Gordon, Daniel W. T. Wundersitz, Jayden R. Hunter, Lisa C. Hanson, Michael I. C. Kingsley

**Affiliations:** 1 Holsworth Biomedical Research Centre, La Trobe Rural Health School, La Trobe University, Bendigo, VIC 3550, Australiaj.hunter@latrobe.edu.au (J.R.H.); l.hanson@latrobe.edu.au (L.C.H.);; 2 Department Rural Allied Health, La Trobe Rural Health School, La Trobe University, Bendigo, VIC 3550, Australia; 3 School of Exercise, Sport and Rehabilitation Sciences, Faculty of Science, University of Auckland, Auckland 1142, New Zealand

**Keywords:** field test, cardiorespiratory fitness, ventilatory threshold, cardiac rehabilitation

## Abstract

The aims of this study are the following: To examine whether field tests predict cardiorespiratory fitness in people with coronary heart disease (CHD) and to determine if heart rate (HR) agreement between the first ventilatory threshold (VT_1_) and field tests is sufficient for prescribing exercise intensity. Participants randomly completed field tests and a cardiopulmonary exercise test (CPET). Linear regression models were developed to predict VT_1_. Agreement between predicted and measured peak oxygen consumption (V̇O_2peak_) as well as field test terminal HR and HR at VT_1_ (VT_1HR_) was assessed using Pearson correlations, Bland–Altman analyses, mean absolute percentage error (MAPE), Lin’s concordance correlation coefficient (CCC), and standard error of estimate (SEE). Agreement between predicted and measured V̇O_2peak_ was modest (Pearson’s r = 0.27–0.77; Lin’s CCC = 0.132–0.735; MAPE = 16.1–30.1%; SEE = 4.7–6.8 mL·kg^−1^·min^−1^). Agreement between field test terminal HR and VT_1HR_ was moderate (Pearson’s r = 0.50–0.67; Lin’s CCC = 0.36–0.68; MAPE = 8.9–13.7%; SEE = 11.9–18.7 bpm; Bland–Altman 95%LOA = −3.5 to 13.7 bpm). Field tests demonstrated variable accuracy for predicting V̇O_2peak_, with none meeting predefined agreement criteria. Regression models indicate field tests can estimate VT_1;_ however, levels of HR agreement indicate CPET is necessary for prescribing exercise intensity.

## 1. Introduction

Symptom-limited cardiopulmonary exercise testing (CPET) with breath-by-breath gas analysis is the criterion measure for cardiorespiratory fitness in cardiac rehabilitation programs [1,2]. Providing precise measurements of peak oxygen consumption (V̇O_2peak_) and identification of physiological thresholds, CPET is essential for risk stratification, individualizing exercise prescription and monitoring adaptation to training [3,4]. In people with coronary heart disease (CHD), prescribing exercise intensity at the first ventilatory threshold (VT_1_) improves cardiorespiratory fitness [1,5,6] while reducing the inter-individual variability in physiological responses observed with range-based prescription [7]. However, the need for specialized equipment and technical expertise limits routine use of CPET, reported in 13% of programs [8], creating a practical barrier to equitable delivery of cardiac rehabilitation. Consequently, there is a need to evaluate alternative approaches for estimating cardiorespiratory fitness and guiding exercise intensity prescription in cardiac rehabilitation settings.

Field tests provide practical, low-cost, and scalable alternatives when CPET is not feasible in cardiac rehabilitation. Commonly implemented tests [4], including the 6 min walk test (6MWT [8,9]), incremental shuttle walk test [10] and Chester Step Test [11], are currently used in clinical practice [4] and have demonstrated utility for estimating V̇O_2peak_ [12,13,14]. A less frequently implemented but clinically relevant option includes the 30 s sit-to-stand, requiring minimal equipment and correlated with V̇O_2peak_ in cancer patients [15,16]. The Astrand-Rhyming Test offers a non-ambulatory, cycle-based alternative, with an established predictive framework [17,18], that aligns with commonly prescribed cycling modalities. While these field tests address the practical constraints across rehabilitation programs, their adoption as viable alternatives to CPET requires robust evidence that they can validly approximate oxygen consumption and inform exercise prescription across heterogeneous cardiac populations.

A linear relationship between heart rate (HR), external workload, and oxygen consumption is assumed when calculating V̇O_2peak_ from field tests. In individuals with CHD, disease-related alterations in cardiovascular physiology may disrupt this linearity, potentially degrading prediction accuracy [19]. Consequently, despite the availability of multiple regression equations, it remains unclear whether commonly used models accurately estimate V̇O_2peak_ in a heterogeneous cardiac rehabilitation population. Establishing their validity in this clinical context is a necessary first step before extending field test applications to more complex outcomes.

The same linearity utilized to predict V̇O_2peak_ may be leveraged to approximate VT_1_ from field test performance and HR responses. However, VT_1_ reflects a ventilatory response rather than a metabolic event and is influenced by disease status, non-humoral regulation [20], exercise protocol characteristics and the inherent subjectivity of threshold identification [19,20]. These factors introduce variability beyond that encountered with V̇O_2peak_, particularly with constant-load or externally paced tests, making VT_1_ prediction more challenging [19]. While integrating ratings of perceived exertion (RPE) may improve VT_1_ estimation in a CHD cohort [12], rigorous evaluation of model accuracy and clinical utility is required before the feasibility of predicting VT_1_ can be determined.

Finally, translating field test estimated thresholds into actionable exercise prescriptions remains difficult without specialized equipment [21] and relies on simple metrics including HR. If field test HR responses reliably correspond to HR at VT_1_ (VT_1HR_), field tests could enable individualized VT_1_-anchored exercise prescription without CPET. However, whether terminal HR from field tests approximate VT_1HR_ closely enough for prescription is uncertain with prior studies limited to 6MWT [22,23] and showing inconsistent results.

The identified knowledge gaps, uncertainty in V̇O_2peak_ prediction accuracy in CHD, limited evidence on field test prediction of VT_1_, and unclear agreement between VT_1HR_ and terminal field test HR, justify further systematic investigation. The aim of this study was three-fold: (1) to assess the validity of existing regression equations for predicting V̇O_2peak_ in a cardiac population; (2) to examine the predictive strength of field test performance outcomes for estimating VT_1_; and (3) to evaluate the level of agreement between VT_1HR_ and terminal HR during field tests.

## 2. Materials and Methods

### 2.1. Design

A single-blind cross-over design with participants completing submaximal field tests in a randomized order was used. The trial protocol was registered with the Australian and New Zealand Clinical Trials Registry a priori (ACTRN12622000875707) with a deviation to include additional measures of agreement between HR metrics including mean absolute percentage error (MAPE), Lin’s concordance correlation coefficient (CCC) [24] and the standard error of estimate (SEE). Project is reported according to the Consolidated Standards of Reporting Trials statement for randomized cross-over trials [25].

### 2.2. Participants

**Figure 1:**
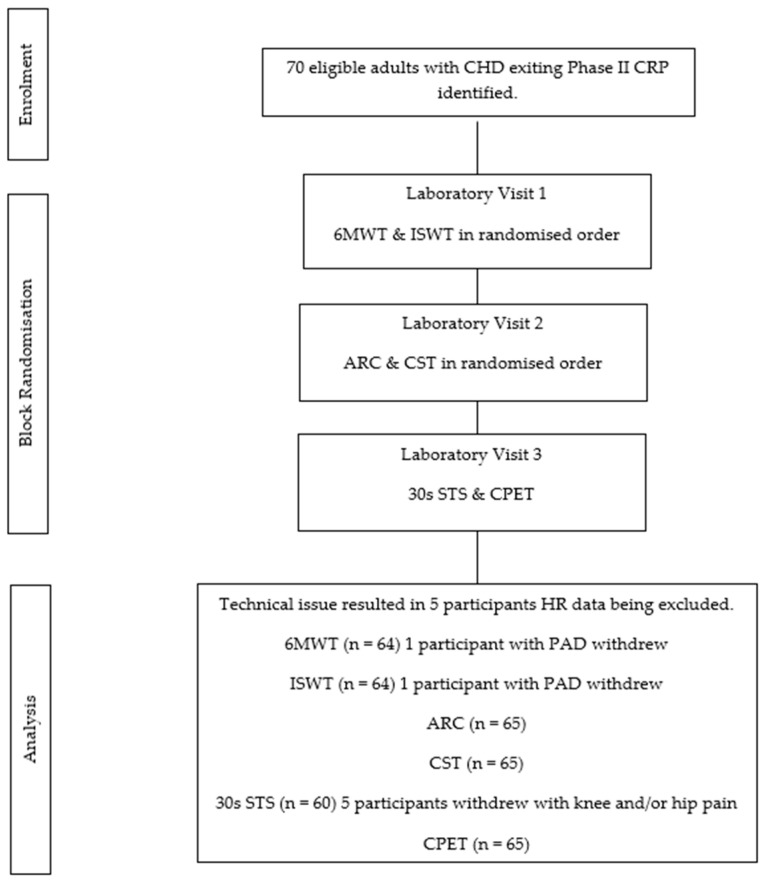
CONSORT Diagram.

Adults, who freely volunteered, were recruited from two regional Australian cardiac rehabilitation centers (Figure 1). Participants were eligible if they were diagnosed and treated for CHD (irrespective of the severity or duration of condition) and attended Phase II cardiac rehabilitation. Participants were excluded if they had a diagnosis of heart failure (left ventricular ejection fraction ≤ 45%), hypertrophic cardiomyopathy, or a prior heart transplant. Additional exclusions included any medical conditions that prevented them from completing exercise testing, and limitations in English language production or comprehension skills that precluded them from understanding the consent form.

### 2.3. Procedures

After providing informed written consent, participants attended three laboratory sessions within seven days (at least 48 h apart) to complete field tests and a symptom limited CPET. Field testing was completed in a block randomization format with the participants completing 6MWT and incremental shuttle walk test in randomized order in the first laboratory visit, Chester Step Test and Astrand-Rhyming Cycle in randomized order in the second laboratory visit before returning to complete the 30 s sit-to-stand and CPET in a third laboratory visit. Heart rate (FT60; Polar Electro Oy, Kempele, Finland) was continuously measured throughout the field tests and the CPET (HRM-W; Garmin, Olathe, KS, USA), with RPE (6–20 Borg Scale [26]) recorded at the completion of each field test and at each stage of the CPET. Exercise tests were terminated if participants self-terminated or reported adverse cardiac symptoms (dyspnea, dizziness, or angina). Both HR and RPE were monitored during rest periods (minimum of 15 min) between tests to ensure adequate recovery.

### 2.4. Laboratory Sessions

#### 2.4.1. Cardiopulmonary Exercise Test

Participants completed the symptom-limited CPET on a cycle ergometer (Excalibur Sport; Lode B. V., Groningen, The Netherlands) using a ramp protocol beginning at 10–30 W and increasing by 10–15 W per minute depending on current activity levels until symptom-limited or volitional exhaustion was reached. Oxygen consumption was measured by indirect calorimetry (COSMED Quark; COSMED, Rome, Italy) with V̇O_2peak_ determined as the highest average oxygen consumption achieved over 30 s. Both HR and RPE were collected at the end of each stage and upon CPET termination with VT_1_ identified in post-analysis using both the v-slope and ventilatory equivalent methods [27].

#### 2.4.2. 6-Minute Walk Test

Participants performing the 6MWT were instructed to cover as much distance in 6 min, in a self-paced manner, on a 30 m indoor walking track with standard conditions, instructions and prompts, according to the American Thoracic Society [9]. The primary outcome measures included the distance covered, measured to the nearest meter using laps completed and interpolation between cones placed at 3 m intervals on the walking course, HR and RPE at the completion of the test.

#### 2.4.3. Incremental Shuttle Walk Test

Participants were required to complete laps of a 10 m walking track marked by two cones placed 0.5 m from each end point on a flat indoor surface. The protocol was externally paced using audio signals at regular intervals, starting at 0.5 m·s^−1^ for the first minute and increasing 0.17 m·s^−1^ each stage. The audio signals indicated when the participant should turn around the cone and begin the next shuttle. The test was terminated if participants could not complete consecutive laps within the time [10]. The primary outcome measures were distance walked, calculated from the number of completed shuttles, peak HR and RPE at termination of the test.

#### 2.4.4. Chester Step Test

Using a step height (initially set at 20 cm and lowered to 15 cm to accommodate knee or hip pain; *n* = 7), participants stepped to a metronome beat of 15 steps·min^−1^ for 2 min, after which HR (FT60; Polar Electro Oy, Kempele, Finland) was recorded. The step rate progressed by 5 steps·min^−1^ per stage every 2 min for a maximum of five stages, participants self-terminated or reached 85% of age-predicted HR maximum [11]. Successful tests were defined as a participant having completed at least two stages. The primary outcome measures were step height, fastest completed step rate, HR and RPE at termination of the test.

#### 2.4.5. Astrand-Rhyming Cycle

Participants cycled on an upright cycle ergometer (Monark 828E, Stockholm, Sweden) at a predetermined resistance (50, 75, 100, 125 or 150 W) based on physical capabilities while maintaining a pedal rate of 50 ± 5 revolutions·min^−1^ for the duration of the test. In healthy populations, an average HR in the final two minutes of the test between 120 and 170 bpm is required to facilitate V̇O_2peak_ prediction [18]. Due to physical deconditioning and the prevalence of HR blunting medication, some participants could not reach or sustain HR between 120 and 170 bpm and had resistance reduced by 25 W to complete the test. Subsequent analysis of cardiorespiratory fitness was therefore based on workload (W), average HR (FT60; Polar Electro Oy, Kempele, Finland) in the 5th and 6th minute and terminal RPE regardless of if participants reached the 120-bpm threshold.

#### 2.4.6. 30 s Sit-to-Stand

Participants, beginning in a standing position in front of the chair, feet pelvis-width apart and arms crossed over the chest, were instructed to perform the sit-to-stand task as many times as possible in 30 s. A repetition was considered successful (and recorded) if participants touched the chair with their thigh or buttocks before returning to the initial position by extending the hips and knees. A demonstration was provided, with the test terminated if the participant required assistance or was unable to complete the movement. No encouragement was provided during the 30 s sit-to-stand test protocol. The chair had no arms, rubber tips on the legs, a hard seat with 46 cm fixed height positioned against the wall [15]. The primary outcome measures were the number of successful repetitions, HR and RPE at completion of the test.

### 2.5. Randomization and Blinding

Testing order was randomly allocated using permuted block, by a computer program (www.randomizer.org) generated by an independent member of the research team and concealed from the investigator enrolling and assessing participants in a sequentially numbered, opaque, sealed envelope. It was not possible to blind participants or researchers to testing order during data collection; members of the research team analyzing the data were blinded to the collection of primary outcome data including testing order.

### 2.6. Sample Size Calculation

An a priori power analysis was performed for multiple linear regression, assuming a medium effect size (Cohen’s f^2^ = 0.15), α = 0.05, power = 0.90, and three predictors (performance measure, terminal HR and RPE), the required total sample size was 67 participants. An a priori power analysis was also performed to determine the sample size required to assess agreement using Lin’s concordance correlation coefficient (CCC). Based on Lin’s CCC_0_ = 0.80 (minimum acceptable agreement) and CCC_1_ = 0.90 (anticipated agreement), with α = 0.05 and 80% power (two-sided), the required total sample size was 60 participants using Fisher’s z approximation [22,24,28]. To meet sample size requirements of both analyses and account for potential dropouts, 70 participants were recruited.

### 2.7. Statistical Analysis

Data distribution was assessed using the Shapiro–Wilk test, and where appropriate, continuous variables are presented as mean ± standard deviation (SD). To evaluate the utility of field tests for predicting VT_1_ (expressed in mL·kg^−1^·min^−1^), multiple linear regression analyses were performed using exercise performance metrics (e.g., time, distance, speed, repetitions), terminal HR, body mass and RPE as predictors. Comparisons between terminal HR from each field test and VT_1HR_ were conducted using paired *t*-tests. The association and agreement between outcomes from field test (predicted V̇O_2peak_ and terminal HR) and criterion measured outcomes (V̇O_2peak_ and VT_1HR_) were examined for relative agreement using Pearson correlation and absolute agreement using Bland–Altman analysis [29]. Limits of agreement (95%LOA) were calculated as mean bias ± 1.96 × SD of the bias. Measurement error was quantified using MAPE, where lower MAPE values indicate better agreement. Accuracy of agreement was further evaluated using Lin’s CCC [24] and the SEE. Acceptable agreement was defined as Lin’s CCC > 0.80 (for both V̇O_2peak_ and VT_1HR_), MAPE ≤ 5% for HR and < 10% for V̇O_2peak_ and SEE ≤ 7 bpm [22] or <3.5 mL·kg^−1^·min^−1^. Statistical significance was set at *p* < 0.05. Analyses were performed using IBM SPSS Statistics (Version 25.0).

## 3. Results

### 3.1. Participant Characteristics and Performance Outcomes

Recruitment took place between August 2022 and June 2025. The 70 participants were predominantly male, aged approximately 70 years, had a primary diagnosis of myocardial infarction or coronary artery disease, had undergone a coronary artery bypass graft and were prescribed multiple medications (Table 1). Performance outcomes for CPET and field tests are reported in Table 2. Equipment malfunction resulted in 5 participants’ data being excluded from field testing agreement analysis.

### 3.2. Prediction of Peak Oxygen Consumption

Predicted V̇O_2peak_ was significantly different to measured V̇O_2peak_ for 6MWT, Astrand-Rhyming Cycle, Chester Step Test and 30 s sit-to-stand t(69) ≥ 4.358, *p* < 0.001. Predicted V̇O_2peak_ was not significantly different to measured V̇O_2peak_ for incremental shuttle walk test *t*(69) = 0.864, *p* = 0.195. There was a large range in the strength of predictability (r = 0.27–0.77, Table 3). Predicted and measured V̇O_2peak_ did not reach a priori levels of agreement for MAPE or SEE (Table 3).

### 3.3. Prediction of First Ventilatory Threshold

Multiple linear regression analyses were conducted to predict VT_1_ (mL·kg^−1^·min^−1^) from performance measures and perceived exertion across different field tests.

6MWT: the model was significant, F(2,67) = 28.99, *p* < 0.001, with an adjusted R^2^ = 0.448, indicating that approximately 45% of the variance in VT_1_ was explained by walking distance (β = 0.040, *p* < 0.001) and RPE (β = −0.766, *p* < 0.001). The prediction equation was:

VT_1_ =3.119 + 0.040 (Distance in m) − 0.766 (RPE).

Incremental shuttle walk test: The model was significant, F(2,67) = 25.22, *p* < 0.001, with an adjusted R^2^ = 0.412, indicating that approximately 41% of the variance in VT_1_ was explained by walking distance (β = 0.018, *p* < 0.001) and RPE (β = −0.857, *p* = 0.005). The prediction equation was:

VT_1_ = 15.55 + 0.018 (Distance in m) − 0.857 (RPE).

Astrand-Rhyming Cycle Test: The model was significant, F(2,67) = 7.93, *p* < 0.001, with an adjusted R^2^ = 0.234, indicating that approximately 23% of the variance in VT_1_ was explained by cycling resistance (β = 0.063, *p* = 0.006) and RPE (β = −0.726, *p* = 0.020). The prediction equation was:

VT_1_ = 17.49 + 0.063 (Resistance) − 0.726 (RPE).

Chester Step Test: The model was significant, F(2,67) = 22.00, *p* < 0.001, with an adjusted R^2^ = 0.477, indicating that approximately 48% of the variance in VT_1_ was explained by step rate (β = 0.670, *p* < 0.001) and RPE (β = −0.860, *p* = 0.002). The prediction equation was:

VT_1_ = 13.298 + 0.670 (Step rate) − 0.860 (RPE).

30 s sit-to-stand: The model was significant F(2,60) = 9.03, *p* < 0.001, with an adjusted R^2^ = 0.277, indicating that approximately 28% of the variance in VT_1_ was explained by number of repetitions (β = 0.624, *p* = 0.001) and change in HR from baseline (β = 0.121, *p* = 0.013). The prediction equation was:

VT_1_ = 9.576 + 0.624 (Chair stands) + 0.121 (ΔHR).

### 3.4. Agreement Between Field Test Terminal and Ventilatory Threshold Heart Rate

**Figure 2:**
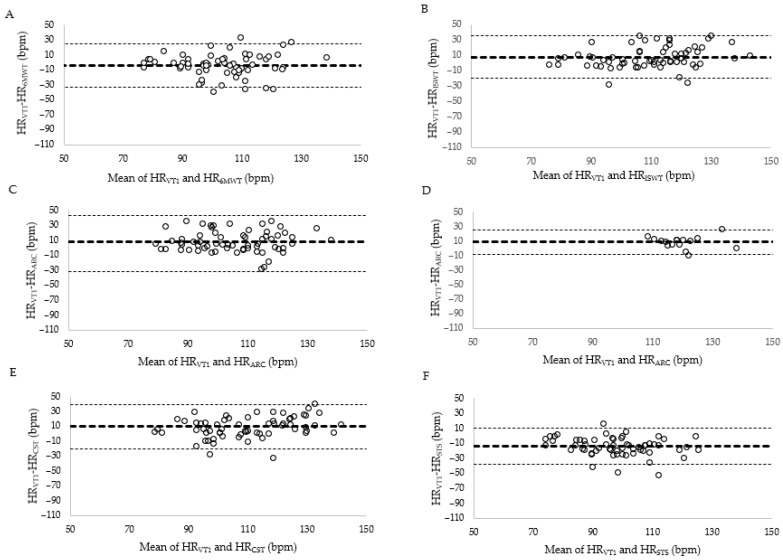
Bland–Altman plot displays the level of agreement between HR at the first ventilatory threshold (VT_1HR_) and HR during the terminal stage of each field test. The middle line represents the mean difference with 95% limits of agreement represented by dashed lines. (**A**) 6 min walk test (6MWT). (**B**) Incremental shuttle walk test (ISWT). (**C**) Astrand-Rhyming Cycle (ARC). (**D**) Astrand-Rhyming Cycle that completed the test (*n* = 16). (**E**) Chest Step Test (CST). (**F**) 30 s sit to stand (STS).

VT_1HR_ was significantly different to terminal HR measured during the incremental shuttle walk test, Chest Step test and 30 s sit-to-stand *t*(64) ≥ 4.773, *p* < 0.001 (Table 4). VT_1HR_ was not significantly different to terminal HR measured during 6MWT *t*(64) = 1.331, *p* = 0.094 or modified Astrand-Rhyming Cycle *t*(64) = 0.798, *p* = 0.214. HR_1VT_ and terminal HR observed during submaximal field test did not reach a priori levels of agreement for CCC (≤0.68), MAPE (≥8.9%) or SEE (≥11.9 bpm; Table 4). Agreement between VT_1HR_ and HR during the terminal stage of each field test is displayed in Figure 2. Beta-blocker status did not have a significant effect on level of agreement between HR_1VT_ and terminal HR observed during submaximal field test CCC (≤0.67), MAPE (≥8.9%) or SEE (≥10.5 bpm; Table 4).

## 4. Discussion

This study evaluated the utility of field tests for measuring cardiorespiratory fitness and guiding individualized exercise prescription in cardiac rehabilitation programs. Predictive accuracy for V̇O_2peak_ did not meet predefined agreement criteria, highlighting limitations of existing prediction equations in individuals with CHD. While regression models built on field test performance and RPE can predict VT_1_, these models may be prone to overfitting and should be applied cautiously in broader cardiac populations. Furthermore, moderate agreement and wide variability between VT_1HR_ and terminal HR observed during field tests suggest that CPET is necessary for prescribing exercise at VT_1_. Collectively, these findings reinforce that CPET with breath-by-breath gas analysis is the gold standard for exercise prescription, with field tests serving as tools to evaluate the effect of cardiac rehabilitation programs on physical function.

In the current study, established predictive equations for estimating V̇O_2peak_ [12,13,14,17] did not meet recommended levels of agreements. While the assumption of a linear relationship between HR, external workload and oxygen consumption in model development provides practical estimations of cardiorespiratory fitness, the complex physiological and clinical characteristics of people with cardiac disease may limit their applicability in practice [19]. In support, the strongest predictive capability was observed with the incremental shuttle walk test equation, which employed a curvilinear exponential model rather than linear approach [13], potentially accounting for the non-linear cardiac response among people with CHD. Methodological differences, including treadmill-based CPET protocols [12], heterogenous cohort (stroke patients for Astrand-Rhyming Cycle) [17] or basing predictions on gas analysis during field testing [13], likely contributed to prediction inaccuracy. These findings underscore that current regression equations for cardiorespiratory fitness have limited generalizability and highlight the need for rigorous validation across diverse cardiac rehabilitation populations before field tests can be confidently recommended for estimating V̇O_2peak_ in clinical practice.

All five field tests demonstrated statistically significant predictive relationships with VT_1_, accounting for 23–49% of the variance. These findings suggest that performance-based metrics have reasonable relative agreement with cardiorespiratory fitness and may serve as alternatives when CPET is unavailable. Incorporating RPE into predictive models enhances accuracy, particularly for individuals with attenuated chronotropic responses due to beta-blocker therapy or autonomic dysfunction [12]. The predictive value of the 6MWT and incremental shuttle walk test was modest compared to previous research [12,30,31], which primarily focused on V̇O_2peak_ developed using treadmill-based CPET [12,30,31] which can yield different VT_1_ values to cycle-CPET employed in the current protocol [32,33]. Further, the characteristics that make VT_1_ an attractive anchor for exercise prescription, being inherently individual and sensitive to both physiological variability and external conditions, also introduce instability when attempting to predict VT_1_ from field tests [19,20]. To our knowledge, the Chester Step Test has not previously predicted VT_1_ among people with CHD; however, it has reported similar predictive utility for V̇O_2peak_ [34]. The modified Astrand-Rhyming Cycle significantly predicted VT_1_ in this study; however, its clinical applicability is limited by deviation from the established protocol, driven by low completion rates within deconditioned participants. Comparable Astrand-Rhyming Cycle completion rates in similar cohorts [17,34] suggest restricted utility in cardiac rehabilitation program settings, regardless of predictive accuracy.

Identifying aerobic workloads equivalent to VT_1HR_ is advised for safe and effective individualization of exercise prescriptions in cardiac rehabilitation programs [3]. The 6MWT, the most commonly used field test within cardiac rehabilitation programs, demonstrated moderate agreement with VT_1HR_ and failed to meet previously reported thresholds for individuals with coronary artery disease on beta blockers [22]. Subgroup analysis by beta-blocker status improved agreement, but still fell short of established benchmarks [22]. Familiarizing participants with field tests is recommended [9] and has been implemented in previous studies, which may have improved agreement levels. However, this approach does not reflect routine clinical practice and may limit the applicability of these tests in cardiac rehabilitation program settings. Despite a larger cohort, our findings align with previous work [30] showing that agreement between VT_1HR_ and terminal HR during the incremental shuttle walk test was insufficient to consider the incremental shuttle walk test a valid surrogate for CPET when prescribing exercise at VT_1HR_ [30]. Consistent with comparable cohorts [22], VT_1_ occurred at approximately 75% of V̇O_2peak_. Given that the incremental shuttle walk test protocol terminates at 85% of age-predicted HR and considering the linear HR–oxygen consumption relationship, it is unsurprising that the average HR at incremental shuttle walk test termination exceeded VT_1HR_.

In the current project, Astrand-Rhyming Cycle was included to facilitate field test assessment among people with CHD with ambulatory limitations and to align with common prescription of stationary cycling in cardiac rehabilitation program. Agreement levels were moderate with poor completion rates similar to those reported in stroke populations [17], limiting clinical applicability in this population group. Reduced resistance to facilitate completion among 75% of participants resulted in a self-paced cycle, which while improving agreement, deviates from the established protocol and prevents direct comparison to earlier studies. Bland–Altman plots and other agreement metrics revealed wide variability across all field tests, indicating potential misclassification of safe workloads if field tests are used in isolation and reinforcing that CPET remains the preferred method for determining VT_1_ [5]. When CPET is unavailable, clinicians can conduct field tests to evaluate physical function and progress but these are not effective for prescribing exercise at VT_1_.

### 4.1. Limitation

Several limitations warrant consideration. First, to reflect clinical practice, a single repetition of each field test was completed by participants. Comparative studies that developed predictive models [12,30,31], in alignment with guidelines [9], completed a familiarization bout of each field test. The current project conducted field tests once to reflect clinical practice, a decision that may have impacted predictive validity in the current project. The small number of participants completing Astrand-Rhyming Cycle at the prescribed resistance limits generalizability and may have biased agreement estimates. Linear regression models trained on specific data sets have the limitation of overfitting data, which may limit generalizability to a diverse cardiac rehabilitation program cohort.

### 4.2. Clinical Implications

These findings have practical relevance for cardiac rehabilitation settings. Field tests provide a moderate approximation of VT_1_ workloads, with varying levels of predictive and prescriptive utility. Terminal HR from field test demonstrated moderate associations with VT_1HR_, supporting utility for group-level analysis. However, at an individual level, self-paced tests (6MWT and 30 s sit-to-stand) tend to underestimate VT_1HR_, while externally paced or fixed workload tests (incremental shuttle walk test, Astrand-Rhyming Cycle and Chester Step Test) tend to overestimate VT_1HR_. Combined with substantial variability in HR agreement, field tests demonstrate limited application for threshold-based prescription. Incorporating RPE into predictive models enhances accuracy, particularly for individuals with attenuated chronotropic responses due to beta-blocker therapy or autonomic dysfunction [12]. In the absence of CPET, cardiac rehabilitation programs may benefit from integrating modified Astrand-Rhyming Cycle protocols or externally paced walking tests (e.g., incremental shuttle walk test); however, clinicians would be encouraged to apply conservative adjustments to intensity monitor patients closely.

## 5. Conclusions

Performance metrics and perceived exertion during ambulatory, functional, and externally paced field tests explained a meaningful proportion of variance in VT_1_, supporting their potential role in estimating cardiorespiratory fitness when CPET is not feasible. However, moderate agreement and wide variability between terminal HR and VT_1HR_ indicate that CPET remains the preferred method for precise exercise prescription in cardiac rehabilitation programs. In settings where CPET is unavailable, the 6MWT and a self-paced modified Astrand-Rhyming Cycle may offer practical alternatives. Further research is needed to validate these approaches across diverse cardiac rehabilitation populations and improve the generalizability of predictive models.

## Figures and Tables

**Table 1:** Demographic and clinical characteristics.

	Total (*n* = 70)	Agreement (*n* = 65)
Age (years)	70.3 ± 7.5	70.5 ± 7.7
Sex (*n*/%)		
Male	46 (66)	43 (78)
Female	24 (34)	22 (22)
Height (cm)	171 ± 9	171 ± 9
Weight (kg)	81.5 ± 17.7	80.8 ± 16.9
Body Mass Index (kg·m^2^)	27.7 ± 5.0	27.5 ± 4.8
Blood Pressure (mmHg)		
Systolic	129 ± 14	130 ± 14
Diastolic	76 ± 9	76 ± 9
Diagnosis (*n*)		
Myocardial Infarction	12	11
Coronary Artery Disease	29	27
Acute Coronary Syndrome	5	5
Coronary Artery Bypass Graft	15	13
Percutaneous Coronary Intervention	11	10
Valve replacement	7	6
Angina	2	2
Medication (*n*)		
Beta Blocker	29	27
Angiotensin-Converting Enzyme Inhibitor	9	9
Statin	51	48
Calcium Channel Blocker	6	5
Angiotensin Receptor Blocker	8	8
Aspirin	49	44
Nitrate	2	1

Regression column includes total population data used in the regression analysis (*n* = 70). Agreement column includes data used in the agreement analysis with 5 participants’ data excluded due to equipment malfunction (*n* = 65).

**Table 2:** Cardiopulmonary exercise and submaximal field test outcomes.

Measure	Analysis Group	
	Regression (*n* = 70)	Agreement (*n* = 65)
CPET		
V̇O_2peak_ (mL·kg^−1^·min^−1^)	20.0 ± 5.8	20.2 ± 5.9
VT_1_ (mL·kg^−1^·min^−1^)	15.1 ± 4.6	15.3 ± 4.7
V̇O_2peak_/VT_1_ (%)	76 ± 8	76 ± 7
HR_peak_ (bpm)	131 ± 22	129 ± 21
VT_1HR_ (bpm)	107 ± 16	105 ± 15
HR_rest_ (bpm)	69 ± 10	69 ± 10
RPE	13 ± 1	13 ± 2
RER	1.1 ± 0.1	1.1 ± 0.1
Peak resistance (W)	130 ± 45	130 ± 45
6 min Walk Test		
Distance (m)	533 ± 79	537± 77
HR (bpm)	102 ± 17	101 ± 16
RPE	12 ± 2	12 ± 2
Incremental Shuttle Walk Test		
Distance (m)	604 ± 174	608 ± 176
HR (bpm)	113 ± 19	112 ± 19
RPE	13 ± 2	13 ± 2
Chester Step Test		
Step Rate	25 ± 5	25 ± 6
HR (bpm)	116 ± 20	115 ± 19
RPE	14 ± 2	14 ± 2
Astrand-Rhyming Cycle Test		
Resistance (W)	65 ± 26	65 ± 26
HR (bpm)	106 ± 16	105 ± 16
RPE	13 ± 2	13 ± 2
30 s Sit-to-Stand		
Repetitions	12 ± 3	12 ± 3
HR (bpm)	92 ± 13	91 ± 13
RPE	12 ± 2	12 ± 2

bpm; beats per minute, CPET; cardiopulmonary exercise test, HR; heart rate, HR_peak_; peak heart rate, HR_rest_; resting heart rate, RER; respiratory exchange ratio, RPE; rating of perceived exertion, VT_1_; first ventilatory threshold, VT_1HR_; heart rate at the first ventilatory threshold, V̇O_2peak_; peak oxygen consumption. Regression column includes total population data used in the regression analysis (*n* = 70). Agreement column includes data used in the agreement analysis with 5 participants’ data excluded due to equipment malfunction (*n* = 65).

**Table 3:** Peak oxygen consumption established prediction.

	Predicted V̇O_2peak_ (mL·kg^−1^·min^−1^)	*p* Value	Equation	Pearsons	Lin’s CCC (95% CI)	MAPE (%)	SEE (mL·kg^−1^·min^−1^)
6 min Walk Test	23.4 ± 4.9	<0.001	[12]	0.70	0.58 (0.40 to 0.71)	25.8 ± 20.4	5.5
Incremental Shuttle Walk Test	20.4 ± 4.4	0.195	[13]	0.62	0.59 (0.42 to 0.72)	16.1 ± 17.2	4.7
Astrand-Rhyming Cycle	17.5 ± 5.2	<0.001	[17]	0.59	0.54 (0.35 to 0.68)	21.3 ± 17.7	5.7
Astrand-Rhyming Cycle Completed (*n* = 16)	20.3 ± 6.9	<0.001		0.77	0.74 (0.61 to 0.83)	20.7 ± 12.9	4.7
Chester Step Test	24.6 ± 5.3	<0.001	[14]	0.70	0.53 (0.34 to 0.68)	30.1 ± 23.6	6.5
30 s sit-to-stand	16.5 ± 2.7	<0.001	[16]	0.27	0.13 (0.07 to 0.38)	22.4 ± 17.3	6.8

Lin’s CCC; Lin’s concordance correlation coefficient, MAPE; mean absolute percentage error, SEE; standard error of estimate. *p* value refers to comparison between predicted and measured V̇O_2peak_. Equation refers to method used to estimate V̇O_2peak_.

**Table 4:** Level of agreement for terminal field test and first ventilatory threshold heart rate.

Field Test	MD ± SD	Pearson Correlation	Lin’s CCC (95% CI)	MAPE (%)	SEE (bpm)
Terminal HR					
6 min Walk Test (*n* = 64)	4 ± 15	0.50	0.50 (0.30 to 0.65)	10.5	15.4
Incremental Shuttle Walk Test (*n* = 64)	−7 ± 14	0.65	0.59 (0.41 to 0.72)	11.3	15.8
Astrand-Rhyming Cycle	1 ± 19	0.67	0.68 (0.53 to 0.79)	8.9	11.9
Astrand-Rhyming Cycle Completed (*n* = 16)	−9 ± 9	0.53	0.36 (0.14 to 0.54)	9.1	11.9
Chester Step Test	−10 ± 15	0.64	0.54 (0.35 to 0.68)	13.5	17.9
30 s sit-to-stand (*n* = 60)	14 ± 12	0.64	0.41 (0.19 to 0.58)	13.7	18.7
Terminal RPE					SEE (a.u.)
6 min Walk Test (*n* = 64)	1 ± 2	0.11	0.09 (−0.14 to 0.32)	13.1	2.4
Incremental Shuttle Walk Test (*n* = 64)	0 ± 2	0.13	0.13 (−0.10 to 0.36)	11.4	1.9
Astrand-Rhyming Cycle	0 ± 2	0.06	0.07 (−0.17 to 0.41)	8.9	2.1
Chester Step Test	−1 ± 2	0.22	0.20 (−0.04 to 0.41)	13.5	2.2
30 s sit-to-stand (*n* = 60)	2 ± 2	0.10	0.07 (−0.16 to 0.30)	13.7	2.7
Terminal HR for participants prescribed Beta Blocker (*n* = 28)					SEE (bpm)
6 min Walk Test	2 ± 12	0.63	0.62 (0.46 to 0.75)	8.9	11.8
Incremental Shuttle Walk Test	−9 ± 15	0.61	0.50 (0.30 to 0.66)	12.6	17.0
Astrand-Rhyming Cycle	−1 ± 11	0.68	0.67 (0.52 to 0.78)	9.7	10.5
Chester Step Test	−10 ± 17	0.56	0.43 (0.23 to 0.61)	14.7	19.1
30 s sit-to-stand	12 ± 11	0.70	0.51 (0.31 to 0.66)	12.0	15.9
Terminal HR for participants not prescribed Beta Blocker (*n* = 37)					
6 min Walk Test	13 ± 16	0.38	0.61 (0.43 to 0.73)	11.6	17.6
Incremental Shuttle Walk Test	−7 ± 13	0.66	0.56 (0.37 to 0.70)	10.3	14.9
Astrand-Rhyming Cycle	1 ± 13	0.64	0.64 (0.48 to 0.76)	8.3	12.6
Chester Step Test	−10 ± 14	0.66	0.55 (0.37 to 0.70)	12.5	16.9
30 s sit-to-stand	16 ± 13	0.56	0.31 (0.08 to 0.50)	15.0	20.7

Lin’s CCC; Lin’s concordance correlation coefficient, MAPE; mean absolute percentage error, SEE; standard error of estimate, RPE; rating of perceived exertion.

## Data Availability

The data that support the findings of this study are available from the corresponding author upon reasonable request.

## Abbreviations

The following abbreviations are used in this manuscript:

CHD	Coronary heart disease
CPET	Cardiopulmonary exercise test
HR	Heart rate
HR_peak_	Peak heart rate
HR_rest_	Resting heart rate
Lin’s CCC	Lin’s concordance correlation coefficient
LOA	Limit of agreement
MAPE	Mean absolute percentage error
RPE	Rating of perceived exertion
SEE	Standard error of estimate
VT_1_	First ventilatory threshold
VT_1HR_	Heart rate at the first ventilatory threshold
V̇O_2peak_	Peak oxygen consumption
6MWT	6 min walk test
